# Structure and Function of the LmbE-like Superfamily

**DOI:** 10.3390/biom4020527

**Published:** 2014-05-16

**Authors:** Shane Viars, Jason Valentine, Marcy Hernick

**Affiliations:** Department of Pharmaceutical Sciences, Appalachian College of Pharmacy, Oakwood, VA 24631, USA; E-Mails: sviars@students.acpharm.org (S.V.); jvalentine@students.acpharm.org (J.V.)

**Keywords:** metallohydrolase, metal-dependent deacetylase, zinc, LmbE-like, PIG-L, MshB, teicoplanin, BshB, mycothiol-conjugate amidase, bacillithiol-conjugate amidase

## Abstract

The LmbE-like superfamily is comprised of a series of enzymes that use a single catalytic metal ion to catalyze the hydrolysis of various substrates. These substrates are often key metabolites for eukaryotes and prokaryotes, which makes the LmbE-like enzymes important targets for drug development. Herein we review the structure and function of the LmbE-like proteins identified to date. While this is the newest superfamily of metallohydrolases, a growing number of functionally interesting proteins from this superfamily have been characterized. Available crystal structures of LmbE-like proteins reveal a Rossmann fold similar to lactate dehydrogenase, which represented a novel fold for (zinc) metallohydrolases at the time the initial structure was solved. The structural diversity of the *N*-acetylglucosamine containing substrates affords functional diversity for the LmbE-like enzyme superfamily. The majority of enzymes identified to date are metal-dependent deacetylases that catalyze the hydrolysis of a *N*-acetylglucosamine moiety on substrate using a combination of amino acid side chains and a single bound metal ion, predominantly zinc. The catalytic zinc is coordinated to proteins via His_2_-Asp-solvent binding site. Additionally, studies indicate that protein dynamics play important roles in regulating access to the active site and facilitating catalysis for at least two members of this protein superfamily.

## 1. Introduction

Metallohydrolases are enzymes that utilize essential metal ion(s) to catalyze the hydrolysis of various biological substrates. In addition to the catalytic metal ion(s), these enzymes also use active site side chains to facilitate the required proton transfer reactions. Since the substrates for these enzymes often carry out important biological functions, several metallohydrolases are targets for drug development. Consequently, there are on-going research efforts focused on enhancing our understanding of the structure and function of specific metallohydrolases to facilitate drug development. A comprehensive review of mononuclear and binuclear metallohydrolases encompassing several protein superfamilies was recently published [1]. This review will not duplicate the scope of that review, but rather will focus on the advances that have been made regarding our understanding of the LmbE-like superfamily of metallohydrolases, a more recently discovered superfamily with a number of interesting drug targets for the development of antibiotics.

## 2. LmbE-like Enzymes

**Figure 1:**
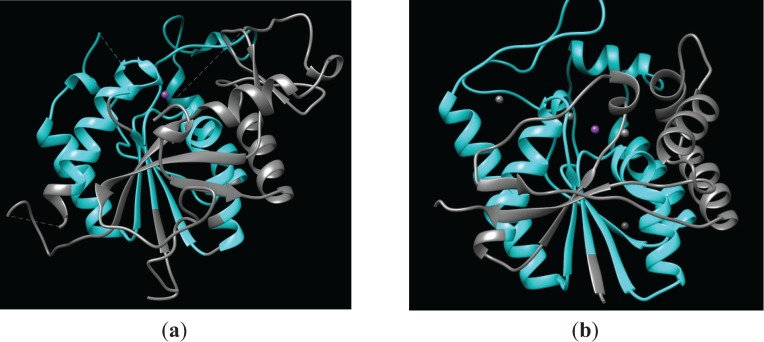
Representative structures of LmbE-like enzymes containing the PIG-L domain. (**a**) Structure of MshB (PDB 4EWL [5]) with the PIG-L domain (residues 8–158) shown in cyan. The catalytic zinc ion is represented as a purple sphere; (**b**) Structure of teicoplanin deacetylase (PDB 3DFM [6]) with the PIG-L domain (residues 11–175) shown in cyan. The catalytic zinc ion is represented as a purple sphere, while non-catalytic zinc ions are represented as gray spheres.

The LmbE-like enzymes are a superfamily of metalloenzymes that are defined by a common PIG-L domain (Pfam [2] PF02585; Table 1). The PIG-L domain name itself comes from the enzyme *N*-acetyl-d-glucosaminylphosphatidylinositol deacetylase (PIG-L), one of the initial members of this family to be identified [3]. Although the structure of PIG-L has not been reported, the structures of five other members of the LmbE-like family have been solved (Table 2), which has aided our understanding of this metalloenzyme superfamily. The overall structure of the LmbE-like enzymes is a single α/β domain that contains at the core a Rossmann fold motif similar to lactate dehydrogenase (Figure 1) consisting of 5–6 β-strands that form a twisted β-sheet surrounded by two pairs of α-helices [4]. This represented a novel fold for a zinc metallohydrolase at the time the first structure was solved. Structurally, the PIG-L domain contains most of the Rossmann fold motif—3–4 of the β-strands, as well as both pairs of α-helices. The PIG-L domains of *N*-acetyl-1-d-myo-inosityl-2-amino-2-deoxy-α-d-glucopyranoside deacetylase (MshB) [5] and teicoplanin deacetylase [6] are shown in cyan (Figure 1), while the catalytic zinc ions are represented as purple spheres. Importantly, this PIG-L domain contains the majority of the catalytically important active site residues, including the side chains that comprise the catalytic metal ion-binding site (Figure 1 and Table 1). In all LmbE-like enzymes characterized to date, the catalytic metal ion has a His_2_-Asp-solvent ligation, which is similar to the metal-dependent deacetylase UDP-3-O-(R-3-hydroxymyristoyl)-*N*-acetylglucosamine deacetylase (LpxC) [1]. This can be further described as a **H**XD_1_**D**_2_ + **H** metal binding motif wherein the two **H** are metal ligands, D_1_ is a general base catalyst (GBC), D_2_ is a metal ligand, and X is any amino acid (Table 1). The distance between metal ligands 1 and 2 is short (2 amino acids), while the spacer region between ligands 2 and 3 is longer and variable (97–148 residues).

**Figure 2:**
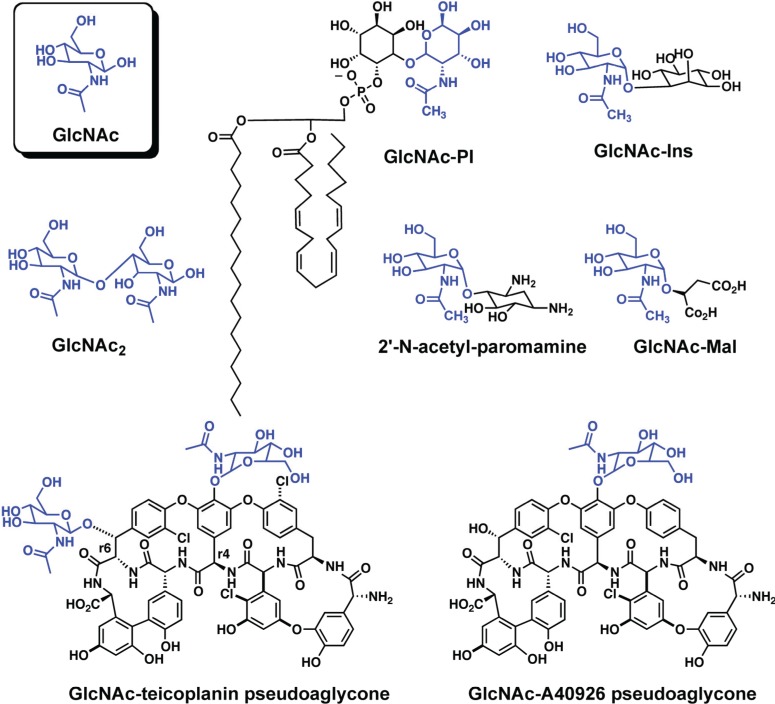
Substrates for LmbE-like enzymes that catalyze deacetylation reactions. The GlcNAc moieties are shown in blue. For molecules that contain more than one GlcNAc moiety, please refer to the text for clarification on the GlcNAc moiety that is cleaved.

**Figure 3:**
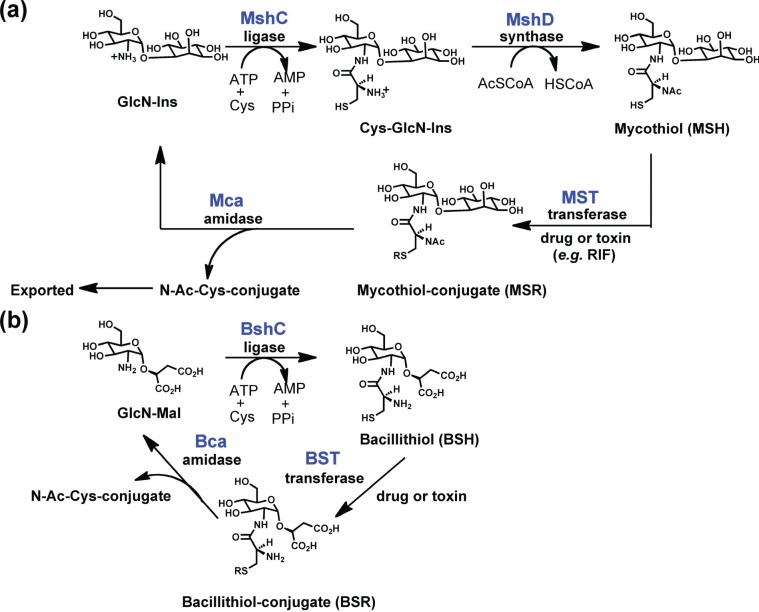
Electrophilic xenobiotic detoxification by (**a**) MSH and (**b**) BSH.

While all of the LmbE-like enzymes identified to date recognize and hydrolyze substrates containing a *N*-acetylglucosamine core, or in case of the enzymes mycothiol-conjugate amidase (Mca) and bacillithiol-conjugate amidase (Bca) a *N*-acylglucosamine core, there is structural diversity amongst the substrates for this enzyme superfamily (Figure 2 and Figure 3) with the *N*-acetylglucosamine core being decorated with various carbohydrate, lipid, and amino acid moieties. This diversity in substrate structure translates into functional diversity with enzymes in the LmbE-like superfamily being responsible for the synthesis of metabolites with important functions, including molecules that serve as protective reducing agents, antibiotics, and cell membrane components. For example, the enzymes MshB and *N*-acetylglucosamine-maleate deacetylase (BshB) are involved in the biosynthesis of mycothiol (MSH; Figure 3) and bacillithiol (BSH; Figure 3), the primary reducing agents in *Mycobacterial* sp. and *Bacillus* sp., [7,8,9] respectively. The enzymes teicoplanin deacetylase and A40926 deacetylase are involved in the biosynthesis of the lipoglycopeptide antibiotics teicoplanin and A40926, while PIG-L is involved in the synthesis of glycosylphosphatidylinositol (GPI) membrane anchors in mammalian and parasitic species [10]. Due to the importance of the metabolites produced by the pathways containing these enzymes, several LmbE-like enzymes are targets for the development of drugs for the treatment of various bacterial and parasitic diseases. Consequently, there is much interest in understanding the structure and function of these enzymes to aid the development of drugs. Herein we review what is known to date regarding the structure and function (*i.e.*, metal binding, mechanism, and molecular recognition properties) of enzymes in the LmbE-like superfamily.

**Table 1:** LmbE-like enzyme conserved PIG-L domains and catalytically important side chains.

Enzyme	UniProtKB Entry *^a^*	PIG-L Domain *^b^*	Metal Ligands *^c^*	Proposed GBC *^c^*
PIG-L	O35790	44–168	His49-Asp52-His157	Asp51
MshB	O50426	8–158	His13-Asp16-His147	Asp15
BshB	Q81ST8	7–124	His12-Asp15-His113	Asp14
Teicoplanin deacetylase	Q6ZZJ1	11–175	His16-Asp19-His164	Asp18
A40926 deacetylase	Q7WZ70	11–172	His16-Asp19-His161	Asp18
TT1542	Q84BR2	5–122	His10-Asp13-His111	Asp12
Mca	L7N5N8	7–152	His12-Asp15-His142	Asp14
Bca	Q81WT0	8–138	His13-Asp16-His127	Asp15
TkDAC	Q6F4N1	35–162	His40-Asp43-His151	Asp42
BtrD	Q4H4F3	9–177	His14-Asp17-His166	Asp16

*^a^* For more information on the UniProt Knowledgebase see [11]. *^b^* For more information on the Pfam database see [2]. *^c^* Assignment based on HXD_1_D_2_ + H motif.

**Table 2:** Available crystal structures of LmbE-like enzymes.

Enzyme	PDB accession code	Bound Ligands *^a^*	Bound Metal Ion	Reference
MshB	1Q74	PE4	Zn	[4]
	1Q7T	BOG, SO4	-	[12]
	4EWL	Act, GOL, PE4	Zn	[5]
BshB (BC1534)	2IXD	Act	Zn	[13]
Teicoplanin deacetylase	3DFF	GOL, PG4	Zn	[6]
	3DFK	DKA	Zn	[6]
	3DFM	SO4	Zn	[6]
	2X9L	BOG	Zn	[14]
	2XAD	BMA, T55, GCS, NAG	-	[14]
A40926 deacetylase	3DFI	-	Zn	[6]
TT1542	1UAN	-	-	[15]

*^a^* Abbreviations: Act, acetate; BMA, β-d-mannose; BOG, β-octylglucoside; DKA, decanoic acid; GCS, d-glucosamine; GOL, glycerol; NAG, *N*-acetyl-d-glucosamine; PE4, polyethylene glycol 4000; PG4, tetraethylene glycol; T55, 8-methylnonanoic acid.

### 2.1. N-acetyl-d-glucosaminylphosphatidylinositol Deacetylase (PIG-L)

PIG-L was one of the first enzymes in the LmbE-like superfamily to be identified [3]. This enzyme catalyzes the hydrolysis of *N*-acetylglucosaminylphosphatidylinositol (GlcNAc-PI; Figure 2) to form glucosaminylphosphatidylinositol (GlcN-PI), an important step in the biosynthesis of GPI membrane anchor precursors found in various mammalian and parasitic species, including *Plasmodium falciparum*, *Trypanosoma brucei*, and *Leishmania major*. For this reason, PIG-L is a target for the development of anti-parasitic agents. PIG-L enzymes have been studied extensively, and were recently the subject of a review [10].

#### 2.1.1. Structure of PIG-L

**Figure 4:**
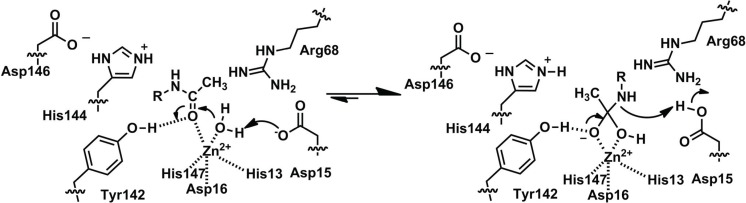
Proposed Mechanism for MshB.

Based on the amino acid sequence of rat PIG-L (rPIG-L), the Pfam [2] PIG-L domain of the protein is made up of residues 44–168 and contains the side chains predicted to make up the catalytic metal ion binding site (Table 1 and Figure 1). The three-dimensional structure of PIG-L has not been reported. However, a three-dimensional homology model of rPIG-L was made using MshB (PDB 1Q7T) as the template and offers insights into PIG-L function [16]. Based on the rPIG-L model the protein ligands for the catalytic zinc ion are proposed to be His49, Asp52, and His157 (Table 1), with 1–2 water molecules likely completing the metal ion coordination sphere. This predicted His_2_-Asp-solvent ligation of the catalytic metal ion is in agreement with the metal ligation of all other known LmbE-like enzymes (Table 1). Results from the biochemical evaluation of the functional importance of the His49, Asp52, and His157 side chains are described below [16]. Based on the active site of the rPIG-L homology model, this enzyme is proposed to catalyze the hydrolysis of the GlcNAc-PI substrate via a carboxypeptidase A-like mechanism [1,16]. The side chain of Asp51 is proposed to function as a GBC to activate the metal-water for attack on carbonyl carbon of substrate, and the resulting tetrahedral intermediate is stabilized by the catalytic metal ion. Next, the now protonated Asp51, or His154, functions as a general acid catalyst (GAC) and transfers a proton to the amine leaving group on substrate to facilitate breakdown of the tetrahedral intermediate. This proposed mechanism shares similarities to the mechanisms proposed for other metallohydrolases [1], including MshB (Figure 4), and has been probed biochemically using site-directed mutagenesis (see below).

#### 2.1.2. Function of PIG-L

The function of PIG-L has been probed biochemically [16,17]. Importantly, rPIG-L activity is abolished following treatment with metal chelators. This inhibition of rPIG-L activity is reversible and is restored upon the addition of divalent metal ions following the trend: Zn^2+^, Cu^2+^ > Ni^2+^ > Co^2+^ > Mg^2+^. The finding that the rPIG-L activity of isolated protein is inhibited by the addition of µM concentrations of Zn^2+^ is consistent with other known metalloenzymes that contain a second (inhibitory) zinc binding site [1]. Importantly, these results confirm that rPIG-L is a metalloenzyme and are consistent with rPIG-L functioning as a mononuclear metalloenzyme. Furthermore, the authors conclude from these studies that rPIG-L is specifically a Zn(II)-dependent enzyme. While this finding is consistent with the reported results, it should be pointed out that Fe^2+^, which activates the LmbE-like enzyme MshB under anaerobic conditions, [18] was not examined in these studies and could also be a potential cofactor for rPIG-L. The proposed ligands for the catalytic metal ion, His49, Asp52, and His157, were also probed biochemically using flow cytometry with cells that were transiently transfected with mutant GFP-fusion proteins [16]. Mutation of these side chains to Ala significantly alters rPIG-L activity in the flow cytometry experiments (magnitude of decreased activity: D52A > H49A > H157A), supporting the proposed roles of these side chains as ligands for the catalytic metal ion. PIG-L from *Entamoeba histolytica* (ePIG-L) does not exhibit the same degree of inhibition by metal chelators as rPIG-L, but the activity of purified enzyme is stimulated by the addition of divalent metal ions (e.g., Mn^2+^, Mg^2+^) [17]. Further studies are needed to probe the basis of the observed differences between rPIG-L and ePIG-L activity.

The proposed mechanism of rPIG-L was also probed using site-directed mutagenesis [16]. In addition to the catalytic metal ion, rPIG-L is proposed to use the side chains of Asp51 and/or His154 as GABC to facilitate the proton transfer reactions in the hydrolysis of the GlcNAc-PI substrate. The importance of the Asp51 and His154 side chains was probed using site-directed mutagenesis using the flow cytometry experiments described above. The D51A mutant has no measurable rPIG-L activity consistent its proposed function as a GABC catalyst, while the activity of the H154A mutant is comparable to WT, suggesting it may not play an important role in the catalytic mechanism under the conditions examined. These studies also identified a number of side chains that may be important for substrate binding [16]. The R88A, D116A, and Q229A mutations had modest affects on PIG-L activity, and based on their position are proposed to contribute to substrate binding. Our understanding of PIG-L would be enhanced by a crystal structure of PIG-L and additional studies aimed at further elucidating the specific roles of side chains in substrate binding and catalysis.

### 2.2. N-acetyl-1-d-myo-inosityl-2-amino-2-deoxy-α-d-glucopyranoside Deacetylase (MshB)

MshB is a metal-dependent deacetylase that catalyzes the hydrolysis of *N*-acetyl-1-d-myo-inosityl-2-amino-2-deoxy-α-d-glucopyranoside (GlcNAc-Ins; Figure 2) to form 1-d-myo-inosityl-2-amino-2-deoxy-α-d-glucopyranoside (GlcN-Ins), an important step in the biosynthesis of MSH (Figure 3) [8]. MSH is the predominant low-molecular-weight thiol in *Mycobacterium* sp. that functions to protect the cell from oxidative damage and also plays a role in the detoxification of xenobiotics [7,8]. Consequently, MshB is a target for antibiotic development. MshB from *M. tuberculosis* and *M. smegmatis* has been characterized to date and the results from these studies are summarized below.

#### 2.2.1. Structure of MshB

**Figure 5:**
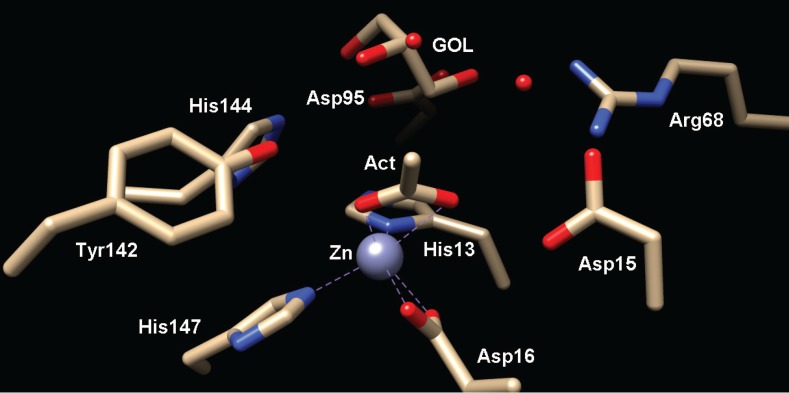
Active site of MshB (PDB 4EWL) containing bound glycerol (GOL) and acetate (Act) molecules. The catalytic Zn^2+^ ion is represented as a gray sphere.

The PIG-L domain of MshB from *M. tuberculosis* is made up of residues 8–158 (Table 1). As mentioned above, the PIG-L domain makes up the majority of the Rossmann fold motif (Figure 1a), and contains the side chains for the metal ion-binding site, as well as most, if not all, of the catalytically important side chains (Table 1). MshB was just the second protein in the LmbE-like superfamily to have its structure solved (Table 2 and Figure 1a) [4]. Importantly, this structure of MshB represented the first structure of a member of the LmbE-like superfamily with known function, as well as the first to contain a bound metal ion. Since the initial structure of MshB was solved, two additional structures have been reported that offer additional insights into MshB function (Table 2) [5,12]. The overall structure of MshB is shown in Figure 1a with the PIG-L domain highlighted in cyan [4]. The N-terminal domain of MshB contains the Rossmann fold motif consisting of five β-strands that form a twisted β-sheet surrounded by two pairs of α-helices, while the C-terminal domain contains two additional β-strands and two α-helices. Two of the available structures of MshB contain a bound zinc ion in the active site [4,5]. The Zn^2+^ ion is coordinated to the active site by the side chains of His13, Asp16, and His147, and has two free sites that can be occupied by water molecules or ligands such as substrate (Figure 5). In the PDB 1Q74 structure, the bound Zn^2+^ ion is pentacoordinate (three protein ligands and two water molecules), [4] while in the 4EWL structure the bound Zn^2+^ ion is tetrahedrally coordinated (4 ligands) [5]. These results likely suggest that the catalytic Zn^2+^ can easily switch between being 4 and 5 coordinate during course of catalysis. This ability to switch coordination numbers one of the attractive features of using Zn^2+^ as a cofactor [1].

Insights into ligand recognition by MshB have been gained from structures of MshB containing bound ligands, as well as docking studies that have been carried out with various substrates and inhibitors [5,12,19,20]. Importantly, these results identify side chains that likely make key binding interactions with the GlcNAc-Ins substrate. Specifically, the side chains of Arg68, Asp95, Tyr142, and His144 are proposed to have hydrogen-bonding interactions with the GlcNAc-Ins substrate [5,12,20]. Additionally, these studies suggest that MshB has at least three mobile loops that play important roles in facilitating substrate binding, chemistry, and product release [5,20,21]. These loops contain some of the key catalytic side chains including Tyr142, which directly participates throughout the MshB catalytic cycle. Docking studies with larger amidase substrates and inhibitors also reveal a hydrophobic cavity adjacent to the MshB active site that may offer an additional site that can be exploited for the development of inhibitors [19,20].

Based on the structures described above, MshB is proposed to catalyze the deacetylation of GlcNAc-Ins through a carboxypeptidase-like mechanism (Figure 4) [4,5] wherein the GlcNAc-Ins substrate is polarized by coordination to catalytic zinc ion and the side chain of Tyr142. Next, the side chain of Asp15 functions as a GBC to activate the metal-water for attack of the carbonyl group on substrate. The resulting oxyanion intermediate is stabilized by the catalytic zinc ion and the side chain of Tyr142. Finally, the now protonated Asp15 serves as a GAC to protonate the amine leaving group and facilitate breakdown of the tetrahedral intermediate. This mechanism has been probed biochemically and the results are described below.

#### 2.2.2. Function of MshB

The activity of MshB is reversibly inhibited by the addition of metal chelators and is restored upon the addition of divalent metal ions to apo-MshB with the following trend: Fe^2+^ > Co^2+^ > Zn^2+^ > Mn^2+^ and Ni^2+^ [18,22]. Additionally, metal titration experiments indicate that the protein is maximally active with 1 metal/protein confirming that the protein is a mononuclear metalloenzyme, [18] consistent with the MshB crystal structures described above. Furthermore, examination of the MshB cofactor preferences reveal that MshB prefers Fe^2+^ when isolated under anaerobic conditions regardless of the metal content of the medium used for protein expression, and switches between Fe^2+^ and Zn^2+^ when purified under aerobic conditions as the metal content of the medium used for protein expression is varied [18]. These results may suggest that MshB can function as a cambialistic enzyme *in vivo*.

The catalytic mechanism of MshB has been probed biochemically using kinetics experiments [21]. Importantly, these studies indicate that the MshB-catalyzed reaction has a bell-shaped dependence on pH (pK_a1_ = 7.3, pK_a2_ = 10.5), consistent with other metallohydrolases. A combination of site-directed mutagenesis and kinetics studies were used to identify the sources of the observed ionizations. Results from these experiments indicate that the side chains of Asp15 and Tyr142 contribute to overall MshB activity and are responsible for pK_a1_ and pK_a2_, respectively, in WT MshB. Furthermore, these results suggest that Asp15 functions as the GBC in the reaction, while Tyr142 likely functions to stabilize the oxyanion intermediate and/or serve as a GAC. These results support the proposed mechanism shown in Figure 4. The importance of specific side chains in substrate binding has also been probed biochemically using site-directed mutagenesis and kinetics experiments [21]. Loss of the His144 and Asp146 side chains, upon mutation to Ala, results in a significant decrease in MshB activity (≤1% WT activity), consistent with a role for these side chains in recognition of substrate. The MshB crystal structures and docking studies described above support a direct role for His144 in the recognition of GlcNAc-Ins, while Asp146 likely works indirectly by interacting with other side chains. Additional studies are needed to further probe the importance side chains in MshB recognition of GlcNAc-Ins in order to identify specific side chains that can be targeted for inhibitor development.

### 2.3. N-acetylglucosamine-maleate Deacetylase (BshB)

BshB is a metal-dependent deacetylase that catalyzes the deacetylation of *N*-acetylglucosamine-maleate (GlcNAc-Mal; Figure 2) to form glucosamine-maleate (GlcN-Mal), an important step in the biosynthesis of BSH (Figure 3) [9]. Similar to MSH, BSH is the primary reducing agent for *Bacillus* sp. and it is also involved in the detoxification of (electrophilic) xenobiotics. Consequently, BshB may be a target for the development of antibiotics. BshB from both *B. anthracis* and *B. cereus* has been characterized to date and the findings from these studies are summarized below [23].

#### 2.3.1. Structure of BshB

The PIG-L domain (residues 7–124), conserved metal ligands (His12-Asp15-His113), and likely GBC (Asp14) for BshB from *B. anthracis* are shown in Table 1. The crystal structure for BshB from *B. cereus* has been solved [13]. At the time the crystal structure was solved, the function of the protein encoded by BC1534 was unknown. However, since then it has been demonstrated that BC1534 encodes for the BshB function in BSH biosynthesis [23]. The N-terminal portion of BshB contains a Rossmann fold motif containing five parallel β-strands surrounded by two pairs of α-helices (similar to Figure 1), which is connected via a loop to the C-terminal portion of the protein that is comprised of two β-strands and two α-helices [13]. The BshB in the crystal is present as a hexamer, and it is thought that the building block of the BshB hexamer is a dimer formed by β-strand exchange, as deletion of the short β8-strand results in inactivation of the enzyme [13]. Importantly, the structure of BshB reveals a bound zinc ion that is tetrahedrally coordinated by the side chains of His12, Asp15, and His113, and one acetate molecule. The acetate molecule likely mimics binding of the *N*-acetyl group on substrate to the catalytic zinc ion. Docking results with GlcNAc-Mal suggest that Arg53 (aligns with conserved Arg68 in MshB) and Arg109 make important contacts with substrate [13]. An overlay of the BshB and MshB enzymes reveals good overlap between the conserved side chains of the metal ligands (BshB: His12-Asp15-His113) and other catalytically important active site side chains (BshB: Asp14, His110, Asp112) [13]. Computational studies suggest that the loops that surround the active site tunnel play an important role in determining access to the active site and the substrate specificity of the enzyme, similar to MshB [24]. Additional insights into ligand recognition by BshB could be gained by obtaining crystal structures of BshB with bound ligands.

#### 2.3.2. Function of BshB

The function of BshB has also been probed biochemically [23,25]. While initial studies of BshB suggested there was no inhibition of enzyme activity following the addition of the metal chelator EDTA, [25] more recent studies demonstrate the ability of this enzyme to be inhibited by treatment with the metal chelator diethylenetriaminepenta-acetic acid [23]. The failure of EDTA to inhibit BshB activity in the initial experiments is suggestive of a tightly bound Zn^2+^ cofactor. The inhibition of BshB by metal chelators is reversible and can be restored upon the addition of divalent metal ions (Zn^2+^, Ni^2+^, Co^2+^) confirming that this enzyme is a metalloenzyme [23]. Furthermore, metal titration experiments indicate that BshB is maximally active with 1 metal/protein, [23] indicating this protein is a mononuclear metallohydrolase. Results from both studies indicate that BshB activity is inhibited by excess Zn^2+^, [23,25] consistent with other metallohydrolases [1].

Due to the similarity of the MshB and BshB active sites, it was thought that BshB catalyzes the deacetylation of GlcNAc-Mal via a mechanism similar to that shown in Figure 4. The BshB mechanism has been probed biochemically using a combination of site-directed mutagenesis and kinetics experiments [23]. Similar to MshB, the BshB-catalyzed reaction has a bell-shaped dependence on pH (pK_a1_ = 6.5, pK_a2_ = 8.5). In contrast to MshB, results from site-directed mutagenesis and kinetics experiments suggest that Asp14 (equivalent to Asp15 in MshB) is responsible for the ionization reflected as pK_a2_ in the pH-profile of WT BshB, suggesting that this side chain functions exclusively as a GAC in the reaction. The pH profiles of Zn^2+^-BshB and Co^2+^-BshB are identical, ruling out the metal-water as source of pK_a1_ in the WT pH profile. Consequently, the mechanism through which BshB catalyzes the hydrolysis of GlcNAc-Mal is not clear. Additional studies are needed to identify source of pK_a1_ in the pH profile and further clarify the roles of specific side chains in the catalytic mechanism. Site-directed mutagenesis and kinetics studies were also used to probe the role of side chains in substrate binding [23]. Results from these studies confirmed the importance of the side chains Arg53, His110, and Arg109 in substrate binding, as loss of these side chains either completely abolishes (R53A) or significantly decreases BshB activity (H110A, R53K, R109K). These findings were expected as both the Arg53 and His110 are conserved across the LmbE-like enzymes (Arg68 and His144 in MshB). Studies on ligand recognition by BshB would be aided by additional crystal structures of BshB containing bound ligands, as well as additional biochemical studies.

### 2.4. Teicoplanin Deacetylase and A40926 Deacetylase

Teicoplanin and A40926 are lipoglycopeptide antibiotics that have shown efficacy against methicillin-resistant *Staphylococcus aureus* (MRSA), vancomycin-resistant *Enterococcus* (VRE), and vancomycin-resistant *S. aureus* (VRSA) with little toxicity [6,14,26]. These antibiotics have a similar mechanism of action to glycopeptides, wherein they target the *N*-acyl-d-Ala-d-Ala peptides of peptidoglycan precursors to inhibit cell wall synthesis in Gram-positive bacteria [6,14,26]. These lipoglycopeptides contain a heptapeptide aglycone that is decorated with *N-*acyl-d-glucosamine, *N-*acetyl-d-glucosamine, and d-mannose. Importantly, the biosynthesis of the lipoglycopeptides requires the deacetylation of the *N*-acetyl-glucosaminyl pseudoaglycones (Figure 2) by teicoplanin deacetylase and A40926 deacetylase prior to attachment of a long chain hydrocarbon that is important for antimicrobial efficacy [6,26,27]. The deacetylation of GlcNAc is regioselective, and it is the GlcNAc moiety attached to the Tyr moiety at the r4 position on the heptamer that is deacetylated [14]. Consequently, there is interest in understanding the enzymes involved in the biosynthesis of these lipoglycopeptides as manipulation of these enzymes may allow for the production of novel lipoglycopeptides that could function as potential antibiotics.

#### 2.4.1. Structure of Teicoplanin Deacetylase and A40926 Deacetylase

**Figure 6:**
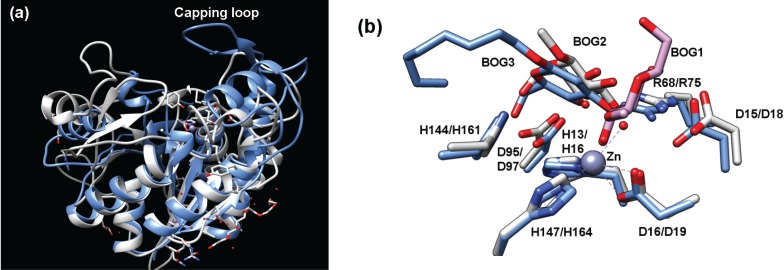
(**a**) Overlay of teicoplanin deacetylase (blue, PDB 2XAD) and MshB (gray, PDB 4EWL) highlights the additional capping loop present in teicoplanin deacetylase; (**b**) Overlay of teicoplanin deacetylase (blue, PDB 2X9L) and MshB (gray, PDB 1Q7T) containing bound BOG molecules. The numbering corresponds to MshB/teicoplanin deacetylase. In this rendering, both MshB monomers are overlaid (BOG1 and BOG2) with teicoplanin deacetylase (BOG3).

The amino acid sequences of teicoplanin deacetylase and A40926 deacetylase suggest that these enzymes are LmbE-like enzymes as they contain PIG-L domains (teicoplanin deacetylase: residues 11–175; A40926: residues 11–172) (Table 1). The overall structures of teicoplanin deacetylase (Figure 1b and Figure 6a) and A40926 deacetylase are similar, as expected based on the 65% sequence identity shared by these enzymes [6,14]. Both proteins possess a single α/β domain made up of nine β-strands and nine α-helices connected by several loops, which includes a Rossmann fold motif containing a twisted β-sheet surrounded by two pairs of α-helices. An important distinction between the pseudoaglycone deacetylases and MshB is the insertion of a large capping loop region (Figure 6a) in teicoplanin deacetylase and A40926 deacetylase, which covers the active site and is located on the opposing end of the hydrophobic, fatty acid binding pocket [6]. The structures of teicoplanin deacetylase (Figure 6b) and A40926 deacetylase each contain a bound pentacoordinate zinc ion that is bound to the protein by the side chains of His16, Asp19, and His164 in teicoplanin deacetylase and His16, Asp19, and His161 A40926 deacetylase (Table 1). The zinc ion coordination sphere in both teicoplanin deacetylase and A40926 deacetylase is completed by two bound solvent molecules. In the teicoplanin deacetylase • decanoic acid (DKA) complex, the carboxylate group of the DKA molecule makes a single interaction with the zinc while one water molecule remains bound to the catalytic zinc ion to maintain the pentacoordinate geometry observed in the above structures [6]. Importantly, the crystal structure of the zinc inhibited teicoplanin deacetylase enzyme has also been solved [6]. This structure reveals that the inhibitory zinc ion is tetrahedrally coordinated to the side chains of His16, Asp97, and Asp163 and a sulfate ion. It is interesting to note that the His16 ligand is shared by both the catalytic and inhibitory Zn^2+^, as the presence of a bridging His in binuclear metal binding sites uncommon [1].

Teicoplanin deacetylase has the largest number of structures available for enzyme•ligand complexes of all the LmbE-like enzymes [6,14]. Structures with bound ligands offer important insights into ligand recognition that can be exploited for the development of therapeutic agents. Based on the available teioplanin deacetylase and A40926 deacetylase structures, it is proposed that the substrate specificity for these lipoglycopeptide deacetylases is determined by a unique, mobile capping loop (Figure 6a) [6,14]. The importance of some of the side chains in the capping loop on enzyme activity is described below. Closer examination of the active site of the teioplanin deacetylaseBOG complex (Figure 6b) suggests that the side chains of Arg75 and Asp97 make important hydrogen bonding interactions with the bound BOG molecule, suggesting they may make similar interactions with bound teicoplanin [14]. Recall that these side chains also make important binding interactions in MshB (overlay Figure 6b). It was initially thought that the DKA molecule in the teicoplanin deacetylase•DKA complex occupied the (anticipated) hydrophobic binding pocket [6], however, it was later shown that the DKA occupies the sugar binding pocket observed in the teioplanin deacetylase•BOG complex and identified the nearby lipid cavity formed by the side chains of V21, L22, L188, P189, Y190, V226, Y229 and L243 [14].

The structures of teicoplanin deacetylase and A40926 deacetylase suggest a mechanism that is highly similar to that proposed for MshB (Figure 4) [6]. First the carbonyl group on substrate binds to the catalytic zinc ion, displacing one of the two bound water molecules. Next, the side chain of Asp18 serves as a GBC to activate the metal water for attack on the carbonyl carbon. The resulting oxyanion intermediate is stabilized by the side chains of His161 and Tyr190 (comparable to His144 and Y142 in MshB). Finally, the now protonated Asp18 serves as a GAC to facilitate breakdown of the tetrahedral intermediate [6]. Additionally, the side chain of Arg75 is proposed to help orient the GBC Asp18, while the side chain of Asp163 is proposed to orient His161.

#### 2.4.2. Function of Teicoplanin Deacetylase and A40926 Deacetylase

The function of teicoplanin deacetylase has been probed biochemically [14,26]. Importantly, the activity of this enzyme is reversibly inhibited by metal chelators and can be restored upon addition of Co^2+^ > Zn^2+^ > Mn^2+^ > Ni^2+^ [14]. These results confirm that teicoplanin is a metalloenzyme. Furthermore, the addition of excess Zn^2+^ inhibits teicoplanin deacetylase activity, consistent with mononuclear metalloenzymes that have neighboring inhibitory Zn^2+^ binding sites [14,26]. These results are supported by the presence of the second (inhibitory) Zn^2+^ site that is observed in the structure that was obtained in the presence of excess zinc. Recall that the catalytic zinc ion is coordinated to the enzyme by the side chains of His16, Asp19, and His164 (Table 1). The functional importance of the His164 side chain has been confirmed biochemically, as the H164N mutant has no measurable teicoplanin deacetylase activity. Additional studies are needed to further clarify the metal ion preferences of both teicoplanin deacetylase and A40926 deacetylase.

Some of the side chains that were identified in the above crystal structures as potentially important for substrate recognition by teicoplanin deacetylase have been probed biochemically using site-directed mutagenesis experiments [14]. Importantly, the Y190F, H161A, and D163N mutants have no measurable activity confirming their importance in the catalytic cycle and consistent with the proposed mechanism described above. Furthermore, the R75Q and D97N mutants also have no measurable activity, consistent with their proposed direct roles in substrate binding. Interestingly, mutations of side chains located in the capping loop and those proposed to be important determinants of substrate specificity lead to either an increase or decrease in activity. Identification of side chains that can be altered to increase the activity of these enzymes is desirable since these enzymes are targets for production of teicoplanin and A40926 analogs.

### 2.5. TT1542

TT1542 is a conserved hypothetical protein that is structurally homologous to other LmbE-like enzymes [15]. To date, the substrate for TT1542 has not been identified, and therefore, the function of TT1542 remains unknown. However, due to the structural homology and sequence similarities of TT1542 with other LmbE-like enzymes, this hypothetical protein likely catalyzes the hydrolysis of a GlcNAc-containing substrate.

#### Structure of TT1542

**Figure 7:**
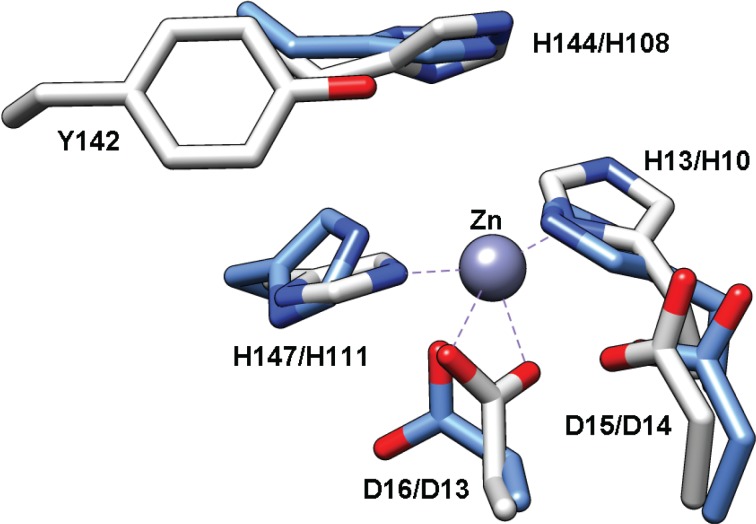
Overlay of TT1542 (blue, PDB 1UAN) with MshB (gray, PDB 4EWL). The Zn^2+^ bound to MshB is represented as a gray sphere. The numbering corresponds to MshB/TT1542.

The PIG-L domain of TT1542 is comprised of residues 5–122 (Table 1). Although the function of TT1542 remains unknown, the crystal structure of this hypothetical protein has been solved and represented the first structure of a protein in the LmbE-like superfamily [15]. Overall, the structure of this conserved protein consists of a twisted β-sheet composed of six parallel β-strands and one antiparallel β-strand, which are all between six α-helices, including a Rossmann fold motif (N-terminus) formed by five β-strands and two pairs of α-helices similar to that observed for other LmbE-like enzymes (Figure 1). Notably, the sixth β-strand and fifth α-helix normally present the Rossmann-fold motif (*i.e.*, GPGTF and NAD(P)-binding superfamilies) are absent in TT1542, [15] which is also consistent with the structures of other LmbE-like enzymes. The C-terminal domain of TT1542 forms a hydrophobic cavity and contains two β-strands and two α-helices (β6-α5-α6-β7) attached to the Rossmann fold motif by a hook-like tail. One unique trait of the TT1542 structure is the positioning of Thr38 at the C-terminus of the β2 strand, which is commonly an acidic residue in most Rossmann fold motifs [15]. Interestingly, there is no bound zinc ion observed in the TT1542 crystal structure. However, an overlay of TT1542 with MshB (Figure 7) suggests that the His10-Asp13-His111 (Table 1) side chains are appropriately positioned to bind the catalytic zinc ion in TT1542. Additional studies are needed to identify the natural substrate for the TT1542 protein. Since the substrate of this protein remains unknown, there are no functional studies reported for this protein.

### 2.6. Mycothiol-Conjugate Amidase (Mca) and Bacillithiol-conjugate Amidase (Bca)

In addition to serving as the primary reducing agents in their respective organisms, MSH and BSH also serve as substrates for transferase enzymes in the detoxification of electrophilic species (Figure 3) [28]. The resulting MS-conjugates (Figure 3) have been shown to be substrates for the metalloamidase Mca, which allows the GlcN-Ins to be recycled back into the MSH biosynthetic pathway while the mercapturic acid is exported from the cell [29]. A functionally similar metalloamidase, Bca, was recently identified that is capable of hydrolyzing BS-conjugates (Figure 3) in *Bacillus* sp. [23]. Presumably, the Bca protein allows for the GlcN-maleate to be recycled. Of note, unlike all the previously described LmbE-like enzymes, which have GlcNAc-containing substrates, Mca and Bca recognize *N*-acylglucosamine containing substrates.

#### 2.6.1. Structure of Mca and Bca

The PIG-L domains of Mca and Bca consist of residues 7–152 (Mca) and residues 8–138 (Bca) (Table 1). While these enzymes are predicted to share structural homology with other LmbE-like enzymes, there are currently no three-dimensional structures of Mca or Bca available, therefore the structures of these enzymes will not be discussed.

#### 2.6.2. Function of Mca and Bca

While most of the work on Mca and Bca has examined the substrate specificity preferences of these enzymes, there has been some initial work done to confirm that these proteins are metalloenzymes. Specifically, it has been shown that purified Mca contains 1.4 Zn^2+^ per enzyme [30]. Furthermore, the activity of isolated Mca is reversibly inhibited by the addition of metal chelators (inhibition 1,10-phenanthroline > 1,7-phenanthroline) and can be restored by the addition of Zn^2+^, consistent with it being a metalloenzyme. Unlike MshB, which shares overlapping substrate specificity with Mca, Mca is inhibited by high concentrations of Zn^2+^ [30]. This suggests that the Mca active site contains differences compared to MshB that allow for the binding of a second (adjacent) Zn^2+^. The Bca protein was only recently identified [23]. Initial attempts to inhibit Bca with EDTA were unsuccessful, although this enzyme is inhibited by high concentrations of Zn [25]. Our understanding of Mca and Bca would be aided greatly by three-dimensional structures of these enzymes and additional studies that probe the metal preferences of these enzymes.

### 2.7. Other Enzymes

Additional LmbE-like enzymes have also been identified, although these enzymes are not as well characterized as those described above. For example, the enzyme BtrD, which is involved in the biosynthesis of butirosin (an aminoglycoside), catalyzes the deacetylation of 2'-*N*-acetyl-paromamine (Figure 2). The amino acid sequence of BtrD suggests that the PIG-L domain (residues 9–177) and catalytically important amino acids, including the metal binding ligands and Asp GBC (Table 1), are conserved [31]. *Thermococcus kodakaraenis* diacetylchitobiose deacetylase (TkDAC), which is involved in chitin degradation, catalyzes the deacetylation of GlcNAc_2_ (Figure 2) [32]. Interestingly, the substrate GlcNAc_2_ also induces the gene cluster for chitin catabolism. The amino acid sequence of TkDAC indicates that the PIG-L domain (residues 35–162) and Asp GBC are conserved (Table 1), suggesting that TkDAC is a LmbE-like enzyme. While the crystal structures of BtrD and TkDAC have not been solved, their amino acid sequences do contain the PIG-L domain (Table 1) suggesting the proteins belong to the LmbE-like superfamily. Further studies are needed to better understand the structures and functions of these proteins.

## 3. Implications and Conclusions

The catalytic metal ion (predominantly Zn^2+^) in all LmbE-like enzymes identified to date is coordinated to the enzyme by a **H**XD_1_**D**_2_ + **H** metal binding motif. In addition to the protein ligands for the catalytic metal ion (shown in bold), this motif also contains a catalytically important side chain that functions as a GBC in all LmbE-like enzymes except for BshB (where this residue is proposed to function as a GAC). Other than BshB, the LmbE-like enzymes appear to catalyze the hydrolysis of substrates through a similar catalytic mechanism (Figure 4). In this mechanism, the carbonyl group on substrate is polarized upon binding to the catalytic metal ion. Next, a nearby Asp serves as a GBC to activate the metal-water for attack on the carbonyl carbon. The resulting oxyanion intermediate is stabilized by the catalytic metal ion, as well as a protein side chain in some enzymes (*i.e.*, Tyr in MshB and teicoplanin deacetylase). The now protonated Asp side chain protonates the amine leaving group to facilitate breakdown of the tetrahedral intermediate. Additionally, studies on the LmbE-like enzymes have identified at least three additional conserved side chains, Arg68, Asp95, and His144 (MshB numbering) that likely play an important role in substrate binding.

The LmbE-like superfamily is a relatively new superfamily of metalloproteins that contains a number of important enzymes. The development of therapeutic agents targeting the LmbE-like enzymes will be aided greatly by information regarding protein structure and function, and the work to date in this area has been summarized in this review. Some of the initial LmbE-like enzymes identified (e.g., PIG-L, MshB) already have reported publications on inhibitor development. As advancements are made in enhancing our understanding of the function of this enzyme superfamily, more inhibitors should be reported. In light of the functional importance of the metabolites produced by the pathways containing the LmbE-like enzymes, this superfamily will continue to be active area of research for years to come.

## Abbreviations

Act: acetate
Bca: bacillithiol-conjugate amidase
BMA: β-d-Mannose
BOG: β-octylglucoside
BSH: bacillithiol
BshB: *N*-acetylglucosamine-maleate deacetylase
DKA: decanoic acid
GAC: general acid catalyst
GABC: general acid-base catalyst
GalNAc: acetylgalactosaminyltransferase
GBC: general base catalyst
GCS: d-glucosamine
GlcNAc: *N*-acetylglucosamine
GlcNAc-Ins: *N*-acetyl-1-d-myo-inosityl-2-amino-2-deoxy-α-d-glucopyranoside GlcNAc-PI, *N*-acetyl-glucosaminylphosphatidylinositol
GlcNAc-Mal: *N*-acetylglucosamine-maleate
GlcN-Ins: 1-d-myo-inosityl-2-amino-2-deoxy-α-d-glucopyranoside
GlcN-Mal: glucosamine-maleate
GlcN-PI: glucosaminylphosphatidylinositol
GOL: glycerol
GPGTF: guanylyl-2',5-phosphoguanosine transferase
GPI: glycosylphosphatidylinositol
LpxC: UDP-3-*O*-(R-3-hydroxymyristoyl)-*N*-acetylglucosamine deacetylase
Mca: mycothiol-conjugate amidase
MRSA: methicillin-resistant *Staphylococcus aureus*
MSH: mycothiol
MshB: *N*-acetyl-1-d-myo-2-amino-2-deoxy-α-d-glucopyranoside deacetylase
NAG: *N*-acetyl-d-glucosamine
PE4: polyethylene glycol 4000
PG4: tetraethylene glycol
PIG-L: *N*-acetyl-d-glucosaminylphosphatidylinositol deacetylase
TB: tuberculosis
TkDAC: *Thermococcus kodakaraenis* diacetylchitobiose deacetylase
TT55: 8-methylnonanoic acid; VRE, vancomycin-resistant *Enterococcus*
VRSA: *vancomycin-resistant Staphylococcus aureus*
WT: wild-type

## References

[1] Hernick M., Fierke C. Mechanisms of metal-dependent hydrolases in metabolism, reference module in chemistry, molecular sciences and chemical engineering. http://mrw.elsevier.com/chem/.

[2] Punta M., Coggill P.C., Eberhardt R.Y., Mistry J., Tate J., Boursnell C., Pang N., Forslund K., Ceric G., Clements J. (2012). The Pfam protein families database. Nucleic Acids Res..

[3] Nakamura N., Inoue N., Watanabe R., Takahashi M., Takeda J., Stevens V.L., Kinoshita T. (1997). Expression cloning of PIG-L, a candidate *N*-acetylglucosaminyl-phosphatidylinositol deacetylase. J. Biol. Chem..

[4] Maynes J.T., Garen C., Cherney M.M., Newton G., Arad D., Av-Gay Y., Fahey R.C., James M.N.G. (2003). The crystal structure of 1-d-myo-inosityl-2-acetamido-2-deoxy-alpha-d-glucopyranoside deacetylase (MshB) from *Mycobacterium tuberculosis* reveals a zinc hydrolase with a lactate dehydrogenase fold. J. Biol. Chem..

[5] Broadley S.G., Gumbart J.C., Weber B.W., Marakalala M.J., Steenkamp D.J., Sewell B.T. (2012). A new crystal form of MshB from *Mycobacterium tuberculosis* with glycerol and acetate in the active site suggests the catalytic mechanism. Acta Crystallogr. D Biol. Crystallogr..

[6] Zou Y., Brunzelle J.S., Nair S.K. (2008). Crystal structures of lipoglycopeptide antibiotic deacetylases: Implications for the biosynthesis of A40926 and teicoplanin. Chem. Biol..

[7] Newton G.L., Buchmeier N., Fahey R.C. (2008). Biosynthesis and functions of mycothiol, the unique protective thiol of actinobacteria. Microbiol. Mol. Biol. Rev..

[8] Hernick M. (2013). Mycothiol, a target for potentiation of rifampin and other antibiotics against *M. tuberculosis*. Expert Rev. Anti Infect. Ther..

[9] Gaballa A., Newton G.L., Antelmann H., Parsonage D., Upton H., Rawat M., Claiborne A., Fahey R.C., Helmann J.D. (2010). Biosynthesis and functions of bacillithiol, a major low-molecular-weight thiol in *Bacilli*. Proc. Natl. Acad. Sci. USA.

[10] Urbaniak M.D., Ferguson M.A.J. (2009). The GlcNAc-PI de-*N*-acetylase: Structure, function, and activity. Enzymes.

[11] Magrane M., Consortium U. (2011). UniProt Knowledgebase: A hub of integrated protein data. Database.

[12] McCarthy A.A., Peterson N.A., Knijff R., Baker E.N. (2004). Crystal structure of MshB from *Mycobacterium tuberculosis*, a deacetylase involved in mycothiol biosynthesis. J. Mol. Biol..

[13] Fadouloglou V.E., Deli A., Glykos N.M., Psylinakis E., Bouriotis V., Kokkinidis M. (2007). Crystal structure of the BcZBP, a zinc-binding protein from *Bacillus cereus*—Functional insights from structural data. FEBS J..

[14] Chan H.-C., Huang Y.-T., Lyu S.-Y., Huang C.-J., Li Y.-S., Liu Y.-C., Chou C.-C., Tsai M.-D., Li T.-L. (2011). Regioselective deacetylation based on teicoplanin-complexed Orf2* crystal structures. Mol. BioSyst..

[15] Handa N., Terada T., Kamewari Y., Hamana H., Tame J.R.H., Park S.-Y., Kinoshita K., Ota M., Nakamura H., Kuramitsu S. (2003). Crystal structure of the conserved protein TT1542 from *Thermus thermophilus* HB8. Protein Sci..

[16] Urbaniak M.D., Crossman A., Chang T., Smith T.K., van Aalten D.M.F., Ferguson M.A.J. (2005). The *N*-Acetyl-d-glucosaminylphosphatidylinositol de-*N*-acetylase of glycosylphosphatidylinositol biosynthesis is a zinc metalloenzyme. J. Biol. Chem..

[17] Ashraf M., Yadav B., Perinthottathil S., Kumar K.S., Vats D., Muthuswami R., Komath S.S. (2011). *N*-Acetyl-d-glucosaminylphosphatidylinositol de-*N*-acetylase from *Entamoeba histolytica*: Metal alters catalytic rates but not substrate affinity. J. Biol. Chem..

[18] Huang X., Kocabas E., Hernick M. (2011). The activity and cofactor preferences of *N*-acetyl-1-d-myo-inositol-2-amino-2-deoxy-α--d-glucopyranoside deacetylase (MshB) change depending on environmental conditions. J. Biol. Chem..

[19] Rogers I.L., Gammon D.W., Naidoo K.J. (2013). Conformational preferences of plumbagin with phenyl-1-thioglucoside conjugates in solution and bound to MshB determined by aromatic association. Carbohydr. Res..

[20] Huang X., Hernick M. (2014). Automated docking studies provide insights into molecular determinants of ligand recognition by *N*-acetyl-1-d-myo-inosityl-2-amino-2-deoxy-α-d-glucopyranoside deacetylase (MshB). Biopolymers..

[21] Huang X., Hernick M. (2012). Examination of mechanism of *N*-Acetyl-1-d-myo-inosityl-2-amino-2-deoxy-α-d-glucopyranoside deacetylase (MshB) reveals unexpected role for dynamic tyrosine. J. Biol. Chem..

[22] Newton G.L., Ko M., Ta P., Av-Gay Y., Fahey R.C. (2006). Purification and characterization of *Mycobacterium tuberculosis* 1d-myo-inosityl-2-acetamido-2-deoxy-a-d-glucopyranoside deacetylase, MshB, a mycothiol biosynthetic enzyme. Protein Expr. Purif..

[23] Fang Z., Roberts A.A., Weidman K., Sharma S.V., Claiborne A., Hamilton C.J., dos Santos P.C. (2013). Cross-functionalities of *Bacillus* deacetylases involved in bacillithiol biosynthesis and bacillithiol-S-conjugate detoxification pathways. Biochem. J..

[24] Fadouloglou V.E., Stavrakoudis A., Bouriotis V., Kokkinidis M., Glykos N.M. (2009). Molecular dynamics simulations of BcZBP, a deacetylase from *Bacillus cereus*: Active site loops determine substrate accessibility and specificity. J. Chem. Theory Comput..

[25] Deli A., Koutsioulis D., Fadouloglou V.E., Spiliotopoulou P., Balomenou S., Arnaouteli S., Tzanodaskalaki M., Mavromatis K., Kokkinidis M., Bouriotis V. (2010). LmbE proteins from *Bacillus cereus* are de-*N*-acetylases with broad substrate specificity and are highly similar to proteins in *Bacillus anthracis*. FEBS J..

[26] Truman A.W., Robinson L., Spencer J.B. (2006). Identification of a deacetylase involved in the maturation of teicoplanin. ChemBioChem.

[27] Ho J.-Y., Huang Y.-T., Wu C.-J., Li Y.-S., Tsai M.-D., Li T.-L. (2006). Glycopeptide biosynthesis: Äâ Dbv21/Orf2* from dbv/tcp gene clusters Are *N*-Ac-Glm teicoplanin pseudoaglycone deacetylases and Orf15 from cep gene cluster is a Glc-1-P thymidyltransferase. J. Am. Chem. Soc..

[28] Newton G.L., Leung S.S., Wakabayashi J.I., Rawat M., Fahey R.C. (2011). The DinB superfamily includes novel mycothiol, bacillithiol, and glutathione S-transferases. Biochemistry.

[29] Newton G.L., Av-Gay Y., Fahey R.C. (2000). A novel mycothiol-dependent detoxification pathway in mycobacteria involving mycothiol S-conjugate amidase. Biochemistry.

[30] Steffek M., Newton G.L., Av-Gay Y., Fahey R.C. (2003). Characterization of *Mycobacterium tuberculosis* mycothiol S-conjugate amidase. Biochemistry.

[31] Truman A.W., Huang F., Llewellyn N.M., Spencer J.B. (2007). Characterization of the enzyme BtrD from *Bacillus circulans* and revision of its functional assignment in the biosynthesis of butirosin. Angew. Chem. Int. Ed..

[32] Tanaka T., Fukui T., Fujiwara S., Atomi H., Imanaka T. (2004). Concerted action of diacetylchitobiose deacetylase and Exo-β-d-glucosaminidase in a novel chitinolytic pathway in the hyperthermophilic archaeon *Thermococcus kodakaraensis* KOD1. J. Biol. Chem..

